# Covariation between plasma phosphate and daytime cortisol in early Parkinson's disease

**DOI:** 10.1002/brb3.556

**Published:** 2016-10-03

**Authors:** Lena Håglin, Lennart Bäckman

**Affiliations:** ^1^ Department of Public Health and Clinical Medicine, Family MedicineUmeå UniversityUmeåSweden

**Keywords:** albumin, cognition, cortisol, Mini‐Nutritional Assessment, Parkinson's disease, phosphate, stress, transferrin

## Abstract

**Background:**

Disturbed phosphate homeostasis in early Parkinson′s disease (PD) may originate from a stress‐related condition and nutritional status among other risk factors, age, and gender.

**Methods:**

Risk of malnutrition using Mini‐nutritional assessment (MNA score) and plasma levels of protein markers and daytime cortisol at the time of diagnosis in PD (*n* = 75) were compared with a control group (*n* = 24). Cognition was assessed using the Mini‐Mental State Examination (MMSE score) and motor function using Unified Parkinson′s Disease Rating Scale (UPDRS‐part III scale).

**Results:**

The patients with PD had significantly lower MNA score than controls which correlated with plasma phosphate levels. The logistic regression revealed that increasing MNA protected from low plasma phosphate, final score (OR = 0.399; 95% CI = 0.196–0.816; *p* = .012) and total score (OR = 0.656; 95% CI = 0.422–1.018; *p* = .060). Phosphate correlated with albumin (*r* = .315; *p* < .006), transferrin (*r* = .331; *p* < .004) and cortisol (*r* = *−*0.355; *p* < .002) confirmed by logistic regressions. Increasing albumin protects from low phosphate after adjusting in logistic regression (OR = 0.806; 95% CI = 0.682–0.952; *p* = .011) and after including variables from Table 1 in backwards elimination, final step (OR = 0.800; 95% CI = 0.660–0.969; *p* = .022). MNA total score and cortisol correlated inversely, confirmed in logistic regression for MNA total score (OR = 0.786; 95% CI = 0.627–0.985; *p* = .037) and for MNA initial score (OR = 0.650; 95% CI = 0.453–0.930; *p* = .020).

**Conclusion:**

This study highlights the importance of phosphate for optimal nutritional status by association with MNA score and albumin in plasma. An inverse relationship between phosphate and cortisol indicate, in addition, that low phosphate levels may affect cognition and motor function in PD.

## 1 Introduction

Studying links between cognition, motor disturbance, and malnutrition in patients with Parkinson′s disease (PD) require understanding of how biomarkers for protein balance and metabolism may be affected by stress and diet. Protein energy malnutrition (PEM) may increase risk of multiple nutrient deficiencies, which in addition to body weight loss and medication, may contribute to both cognition deficits and motor disturbances in PD.

Patients with PD have higher blood cortisol levels than controls, associated with an increased acrophase, increased amplitude, and increased area under the curve of cortisol (Charlett et al., 1998; Hartman, Vedldhuis, Deuschle, Standhardt, & Heuser, 1997). In PD, nonmotor as well as motor symptoms can result from dysregulation within the HPA‐axis, including high levels of cortisol that can have deleterious effects on multiple organ systems. Acute levodopa medication can result in low HPA‐axis activity with a decrease in cortisol levels, most pronounced in patients with severe disabilities (Müller, Welnic, & Muhlack, 2007).

As cortisol is a phosphaturic hormone, loss of phosphate may be the cause of low plasma levels and/or body depletion of high‐ energy phosphate in intracellular compartments associated with PD.

Phosphate depletion may cause anorexia, weight loss, and disturbed energy metabolism with hypoxia in neurological and neuropsychiatric diseases and symptoms (Håglin, 2016). A schematic model for stress‐induced PD suggests that glucocorticoids can be neurotoxic (Smith, Castro, & Zigmond, 2002). Glucocorticoids and chronic stress influence the progress of PD by putting neurons in an energetically unfavorable condition (Kibel & Drenjančević‐Perić, 2008). Salivary cortisol concentration is decreased in patients with PD after tactile massage, a finding that suggests a connection between stress and cortisol levels in this patient population (Törnhage et al., 2013). Salivary cortisol correlates with risk behavior in PD (Djamshidian et al., 2011).

In this study, we examine associations between nutritional status, phosphate, albumin, and transferrin together with cortisol levels, cognition (Mini‐Mental State Examination [MMSE] score), and motor function (Unified Parkinson′s Disease Rating Scale [UPDRS]‐part III scale) for patients with Parkinson's disease by Pearson′s correlations and with logistic regressions.

## 2 Patients and Methods

### 2.1 NYPUM population

This study's population originates from the NYPUM project (NY [new] Parkinsonism in UMeå), a prospective study that focuses on idiopathic forms of Parkinsonism in northern Sweden (Västerbotten county; 142,000 inhabitants; Linder, Stenlund, & Forsgren, 2010). All suspected cases of idiopathic Parkinsonism were referred to the neurological department and this department employed all the neurologists involved in this investigation. From January 2004 to April 2009, 186 cases with Parkinsonism were identified. To confirm the presence of PD, another specialist in movement disorders (blinded to the assessment of the previous examiner) evaluated a videotape of the patient undergoing the UPDRS‐part III scale examination. A patient was included if both examiners judged that the patient had fulfilled the clinical criteria for PD according to the UK Parkinson's Disease Society Brain Bank criteria (Gibb & Lees, 1988). Patients fulfilling diagnostic criteria for PD were included in the NYPUM study. A control group (*n* = 30) was selected by announcements, according to the age and gender of the first 50 patients diagnosed in the NYPUM study.

The present substudy included patients with PD (*n* = 75) and controls (*n* = 24) who took part in the nutritional status assessment at baseline of the NYPUM project. In addition, the baseline data included analysis of nutrition‐related biomarkers in a blood sample (Table 1).

**Table 1:** Patient baseline characteristics and plasma variables in mean (min–max) and with *p*‐values for difference between PD patients and controls

Variables	PD*N* = 75	Controls*N* = 24	*p*‐ValuePD vs. controls
Age (years)	68.9 (46.7–86.8)	68.2 (48.4–75.8)	.653
Women/men (*N*)	34/41	12/12	.690
BMI (kg/m^2^)	26.2 (19.4–40.8)	25.5 (16.8–36.9)	.539
Hoehn and Yahr scale stage, median	2.03 (1.0–3.0)	NA	NA
UPDRS total scale	34.6 (8–63)	NA	NA
UPDRS‐part III scale	24.9 (5–50)	NA	NA
MMSE score	28.7 (24–30)	29.1 (28–30)	.248
MNA—initial score	12.5 (6–14)	12.8 (9–14)	.459
MNA—final score	**13.1 (10.0**–**16.0)**	**14.5 (12.5**–**15.5)**	**.000**
MNA—total score	**25.5 (17.5**–**30.0)**	**27.2 (24.0**–**29.5)**	**.005**
P‐Albumin (g/L)	40.9 (32.2–48.6)	39.3 (33.6–45.3)	.058
P‐Transferrin (g/L)	2.24 (1.56–3.59)	2.27 (1.45–3.40)	.752
P‐Cortisol (nmol/L)	386 (148–779)	335 (147–610)	.113
P‐Phosphate (mmol/L)	1.20 (0.73–1.53)	1.23 (0.97–1.48)	.441

BMI, body mass index (kg/m^2^); UPDRS, Unified Parkinson's Disease Rating Scale; MMSE, Mini‐Mental Examination Score; MNA, Mini‐Nutritional Assessment; NA, not available. Significant values indicated in bold.

### 2.2 Disease severity

Disease severity (i.e., the motor symptom severity) was assessed using the UPDRS total score and UPDRS‐part III scale (Fahn, Elton, &, The UPDRS Development Committee, 1987) and the Hoehn and Yahr staging scale (Hoehn & Yahr, 1967). The UPDRS scoring was performed when the patients were in the ON‐phase and when they started the dopaminergic treatment. The results from the UPDRS‐part III scale were used to study associations with nutritional status, biomarkers, and cognition. Cognitive function was evaluated using the MMSE score, a screening instrument for global cognition, including orientation in time and place, ability to follow simple commands, registration, attention, calculation, memory, naming, writing, and figure copying (Folstein, Folstein, & Hugh, 1975). MMSE score ranges from 0 to 30, with higher scores indicating better cognitive functioning.

### 2.3 Anti‐Parkinson medication

The levodopa equivalent dose (LED) was calculated at baseline and at follow ups, by use of a conversion factor for each of the anti‐Parkinson medication (Tomlinson et al., 2010). Information about LED at baseline revealed that only two patients had started anti‐Parkinson treatment at the time of diagnosis. At first follow up at 6 month, all but nine patients had started treatment and about 50% of the patients had a LED <200 and 8% had >300.

The medication was introduced over the first weeks after diagnosis. The nutritional investigation, including the blood sampling, was performed in a nonstandardized manner according to drug‐therapy during this time period. We found that 13 of the patients started anti‐Parkinson medication prior to the blood sampling, whereas 60 patients started afterward. The mean levels of plasma cortisol in these groups were 412 ± 165 nmol/L (*n* = 13) and 385 ± 139 nmol/L (*n* = 60), respectively; *p* < .537.

### 2.4 Mini‐Nutritional Assessment

The Mini‐Nutritional Assessment (MNA) score, an international validated screening tool, was used to assess nutritional status and was performed when anthropometrical measurements were gathered by the dietician. The screening consists of 18 questions regarding anthropometry, diet, and health (Guigoz, 2006). MNA—total scores between 24 and 30 points indicate optimal nutritional status, MNA total scores between 17 and 23.5 indicate a risk for malnutrition, and MNA scores <17 indicate malnutrition. Initial questions refer to MNA—initial (MNA‐SF; short form) in this study with score from 7 to 14 points. The last items of the MNA score included questions about food, self‐perceived health, and anthropometry (MNA final score). The scores for this information were between 10 and 16 points.

### 2.5 Biochemical analysis

The blood was collected between 8 a.m. and 4 p.m. on the day when the patients were admitted for assessment of nutritional status, dietary intake, and neuro‐psychological tests, corresponding to 2–6 weeks after diagnosis. The collection of nutritional data including the blood sampling was performed before initiation of anti‐Parkinson medication in the majority of the patients (*N* = 60 patients).

The plasma samples were used to measure phosphate, albumin, transferrin, and cortisol levels. The levels of cortisol thus correspond to daytime levels and not early morning or evening levels. Analysis was performed using clinical routines in an accredited laboratory at Umeå University hospital. Plasma cortisol levels were analyzed by Elecsys Cortisol reagent on a Cobas e 601 analyzer. Plasma phosphate, albumin, and transferrin levels were analyzed using Vitros PHOS Slides, Vitros ALB Slides, and Vitros TRFRN reagent, respectively, on an Ortho Vitros 5.1 FS analyzer.

### 2.6 Data analysis

An independent two‐tailed test was used to compare patients and controls. For non‐normal distributed data, a chi‐square test was used. Bivariate correlations were analyzed using the Pearson's correlation coefficient (Tabachnik & Fidell, 2001). A multiple logistic regression was performed with variables from Table 1 included and with low or high phosphate and high and low cortisol as the dependent variable. Backwards elimination was performed with a logistic regression for low or high plasma phosphate with variables included from Table 1. Results at the last step are reported (Table 4). The program Predictive Analytics Software (PASW Statistics, version 18.0.3 SPSS Inc., Chicago IL, USA) was used for the analysis.

The study was approved by the Ethics Committee of the Faculty of Medicine at Umeå University, Sweden (03‐387; 05‐077M; 11‐334‐31M). Written informed consent was obtained from all participants.

## 3 Results

### 3.1 Baseline variables and bivariate correlations

Table 1 shows baseline data in mean (min–max) levels for PD patients (*n* = 75) and controls (*n* = 24) at the time of diagnosis. MNA total and MNA final score was significantly lower in the PD patients compared with the controls (*p* < .005 and <.002, respectively). Table 2 shows the bivariate correlations for plasma levels of albumin, transferrin, cortisol, phosphate, MNA total score, MMSE score, and UPDRS‐part III scale for PD patients (*n* = 75). Phosphate correlated positively with albumin and transferrin (*r* = .315; *p* < .006; *r* = .331; *p* < .004, respectively), and negatively with cortisol (*r* = −.355; *p* < .002) in the PD patients.

**Table 2:** Correlation, (*r*) and *p*‐values between plasma levels and clinical assessments at baseline for patients (*N* = 75) with Parkinson′s disease

PD patients	Albumin	Transferrin	Cortisol	Phosphate	MNA total score	MMSE score
UPDRS III score	−.205	−.086	**.312**	−.186	−.196	−**.349**
	.080	.466	**.007**	.112	.102	**.002**
MMSE score	.109	.027	−.058	**.252**	**.227**	
	.351	.817	.618	**.029**	**.056**	
MNA— total score	**.278**	.111	**−.299**	−.057		
	**.018**	.351	**.011**	.637		
Phosphate	**.315**	**.331**	**−.355**			
	**.006**	**.004**	**.002**			
Cortisol	−.140	**−.252**				
	.232	**.029**				
Transferrin	**.294**					
	**.010**					

UPDRS, Unified Parkinson's Disease Rating Scale; MMSE, Mini‐Mental Examination Score; MNA, Mini‐Nutritional Assessment; NA, not available. Significant values indicated in bold.

Bivariate correlations between MNA total score, MMSE score, and UPDRS‐part III scale are presented in Table 2. Disease severity assessed by UPDRS‐part III scale was negatively correlated with MMSE scores (*r* = −.349; *p* < .002). The MMSE score correlated with MNA score (*r* = .227; *p* < .056) and with plasma phosphate (*r* = .252; *p* < .029) in PD patients. The only correlations shown in the control group was between MMSE score and plasma phosphate (*r* = .449; *p* < .032) and between albumin and transferrin (*r* = .416; *p* = .043). The UPDRS‐part III scale correlated with cortisol in PD patients (*r* = .312; *p* < .007). Bivariate correlations between MNA total score and plasma albumin (*r* = .278; *p* = .018), cortisol (*r* = −.299; *p* < .011) and MMSE score (*r* = .227; *p* < .056) in the PD patients (Table 2).

### 3.2 Multiple logistic regressions

Adjustments were done for age and gender in the two models with either plasma phosphate (low level = 1; 0.73–1.20 and high level = 0; 1.21–1.53) or plasma cortisol (high level = 368.7–778.5 and low level = 147.6–366.8) as the dependent variable (Table 3).

**Table 3:** Logistic regressions with low (0.73–1.20) and high (1.21–1.53) levels of phosphate as the dependent variable (left) and adjusted for age and gender. High (368.7–778.5) and low (147.6–366.8) levels of cortisol (right) adjusted for age and gender. Patients, both women and men with PD included (*N* = 75)

Variables	Phosphate		Cortisol	
	OR (95% CL)	*p*‐Value	OR (95% CL)	*p*‐Value
BMI (kg/m^2^)	1.045 (0.922–1.183)	.493	0.921 (0.816–1.039)	.179
Hoehn and Yahr scale stage	0.717 (0.307–1.672)	.441	0.915 (0.423–1.979)	.822
UPDRS‐part III scale	1.012 (0.962–1.066)	.635	1.020 (0.974–1.069)	.399
UPDRS total scale	1.019 (0.975–1.065)	.406	1.009 (0.969–1.050)	.669
MMSE score	0.958 (0.653–1.405)	.826	1.115 (0.783–1.588)	.547
MNA—initial score	1.015 (0.741–1.389)	.927	**0.650 (0.453**–**0.930)**	**.020**
MNA—final score	0.779 (0.556–1.093)	.148	0.910 (0.671–1.233)	.542
MNA—total score	0.901 (0.725–1.120)	.348	**0.786 (0.627**–**0.985)**	**.037**
Albumin (g/L)	**0.806 (0.682**–**0.952)**	**.011**	1.024 (0.897–1.170)	.721
Transferrin (g/L)	0.227 (0.041–1.250)	.088	0.281 (0.061–1.293)	.103
Cortisol (nmol/L)	**1.004 (1.00**–**1.008)**	**.031**	NA	NA
Phosphate (mmol/L)	**NA**	**NA**	**0.024 (0.001**–**0.830)**	**.039**

BMI, body mass index (kg/m^2^); UPDRS, Unified Parkinson's Disease Rating Scale; MMSE, Mini‐Mental Examination Score; MNA, Mini‐Nutritional Assessment; NA, not available. Significant values indicated in bold.

The results revealed that increasing cortisol is a risk for low phosphate, 1.004, (1.00–1.008) (*p* < .031) while increasing albumin protects from low phosphate (0.806, (0.682–0.952; *p* < .011). The results, with high or low plasma cortisol as the dependent variables, revealed that increase in MNA, initial and total score protected against high cortisol levels (*r* = .650: 0.453–0.930 *p* < .020 and *r* = .786; .627–.627–.985, *p* < .037, respectively). With low or high phosphate as the dependent variables, revealed that increasing phosphate protected against high cortisol levels (0.024:0.001–0.830; *p* < .039; Table 3).

### 3.3 Backwards elimination

The final step of backwards elimination reveals that increasing cortisol increases risk for low phosphate while high albumin decreases risk of low phosphate (Table 4). Gender and MNA final score was also confirmed in this regression. To be women and to increase MNA score, protects from low phosphate levels (Table 4).

**Table 4:** Variables included from Table 1 for PD patients in a multiple logistic regression with either plasma phosphate (low level = 1; 0.73–1.20 and high level = 0; 1.21–1.53) or plasma cortisol (high level = 1; 368.7–778.5 and low level = 0; 147.6–366.8) as the dependent variable. Significant results presented from the last step in backwards elimination (OR with 95% CL) and *p*‐value

	Phosphate		Cortisol	
	OR (95% CL)	*p*‐Value	OR (95% CL)	*p*‐Value
Gender (0 = women, 1 = men)	0.119 (0.031–0.461)	.002		ns
UPDRS total scale		ns	0.839 (0.706–0.997)	.046
UPDRS‐part III scale		ns	1.226 (0.998–1.506)	.053
MNA—final score	0.399 (0.196–0.816)	.012		ns
MNA—total score	0.656 (0.422–1.018)	.060	0.686 (0.515–0.915)	.010
Albumin (g/L)	0.800 (0.660–0.969)	.022	1.237 (1.018–1.503)	.033
Cortisol (nmol/L)	1.008 (1.003–1.014)	.005	NA	NA
Phosphate (mmol/L)	NA	NA	0.001 (0.000–0.114)	.005

UPDRS, Unified Parkinson's Disease Rating Scale; MNA, Mini‐Nutritional Assessment; NA, not available.

The final step of backwards elimination reveals that increasing albumin or UPDRS‐part III scale, increases risk for high cortisol while increasing MNA total score, plasma phosphate or UPDRS‐total decreases this risk (Table 4).

## 4 Discussion

**Figure 1:**
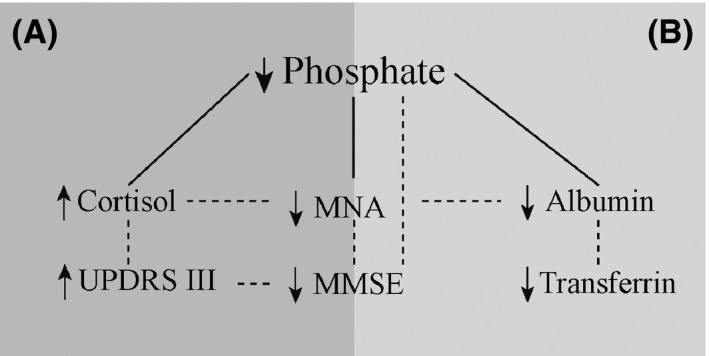
Low plasma phosphate has consequences for energy metabolism in almost all cells in a body and thereby a limiting factor for optimal cognition and motor function. The data presented in Table 2 indicates that the interactions and possible sequences of these events are correlated and can be expressed as two theories; (A) stress‐related decrease in phosphate and transferrin induced by cortisol and (B) protein energy malnutrition with decrease in phosphate and albumin revealed by low MNA score. Plasma biomarkers may explain disease severity assessed with UPDRS‐part III scale and MMSE score, respectively, through low plasma phosphate level

The plasma phosphate concentration was positively associated with plasma albumin, transferrin, and MNA score at the time of diagnosis, indicating an involvement in nutritional status (Fig. 1). The results from this study indicates that covariance between phosphate and cortisol, may reveal adverse clinical outcome, including low MMSE with low phosphate and high UPDRS‐part III scale with high cortisol. Phosphate decreases with stress, explained by concomitant increase in cortisol in post‐traumatic conditions (Bech, Blans, Telting, & De Boer, 2013). An altered protein metabolism in PD patients may also be associated with high cortisol levels—a stress‐induced catabolic response frequently reported as weight loss. Increase in cortisol may exacerbate disease progress by loss of weight and skeletal muscle and increased visceral fat and thereby cause sarcopenic obesity. In a study on gait difficulties in PD, the level of cortisol was similar to the level found in the present patient population (Charlett et al., 1998). This finding can be either due to a stress‐related disturbance in Parkinsons′s disease, shown by higher levels of cortisol, that causes low phosphate levels or be due to malnutrition with disturbed protein status with high cortisol.

Low albumin and low phosphate have been shown to be associated in geriatric patients (Kagansky, Levy, Koren‐Morag, Berger, & Knobler, 2005) and in a healthy population (Wojcicki, 2013). Thus, a low level of phosphate may affect protein metabolism negatively. It has been reported that visceral proteins could be a useful index in detecting malnutrition among elderly (Kagansky et al., 2005; Sergi et al., 2006). In clinical practice, transferrin may be used as an indirect marker of PEM, but transferrin is influenced by many conditions associated with old age and in this study, negatively associated with plasma cortisol in the bivariate analysis. Transferrin may respond as an acute phase reactant (short term compared with albumin) and thus also be an indication of acute severe condition (stress) rather than chronic malnutrition. In addition, transferrin increases with iron deficiency, a condition present in some cases of malnutrition (Ingenbleek, Van Den Schrieck, De Nayer, & De Visscher, 1975).

Low phosphate levels has been neglected in clinical studies, as an important biomarker for malnutrition, whereas low levels of albumin and transferrin have been used in prognostic indexes of chronic malnutrition and transferrin as a marker for negative nitrogen balance and visceral protein depletion (Ingenbleek et al., 1975). Independent of causes, low phosphate levels have detrimental clinical outcome in diseases and is associated with morbidity and mortality among elderly with a long hospital stay and lower albumin levels (Kagansky et al., 2005; Sumukadas et al., 2009).

Phosphate and its importance for energy generation, anabolism, and acid‐base maintenance have been neglected with respect to studies in neurological diseases. Brain bioenergetics has impact on cognitive ability and various Pi containing ratios correlated with cognition in both adults and children/adolescents (Heinze et al., 2015). Hattingen et al. demonstrated mitochondrial dysfunction in early PD (Hattingen et al., 2009). Using PET scanning and metabolic ratio, Liepelt et al. (2009) found an association between hypometabolism and both UPDRS‐part III scale and MMSE score and concluded that cerebral hypometabolism in PD is primarily associated with cognitive impairment.

It has been shown in an experimental study that stress by high cortisol exaggerates motor symptoms in a rat model of PD (Smith, Jadavji, Colwell, Perehudoff, & Metz, 2008). Clinical studies have reported that high levels of cortisol can have negative effects on some cognitive functions that are measured during day time (Comijs et al., 2010). A high level of the MMSE score was associated with high plasma phosphate and high MNA score, indicating good nutritional status and high level of cognition with low cortisol levels during daytime. However, among elderly, low cortisol levels in the evenings are associated with depression indicating influence from diurnal variation on these associations (Bremmer et al., 2008; Kabia, Rhebergen, Van Exel, Stek, & Comijs, 2016).

On the contrary, the negative relationship between UPDRS‐part III scale and MMSE‐ score in the logistic regression (Table 3) and between UPDRS‐part III scale and cortisol, MMSE and phosphate (Table 2) may reveal adverse effects and some mechanism behind the covariance between phosphate and cortisol on motor dysfunction (Fig. 1A). It has been shown that acute L‐dopa administration induces a decrease in cortisol by a decreased function of HPA‐axis which is more pronounced in advanced stage of the disease (Müller et al., 2007). Patients who started anti‐Parkinson medication before the blood sampling for cortisol analysis did not have lower cortisol than patients who received the medication after cortisol assessments. Thus, the anti‐Parkinson medication was not a confounder for conclusions about negative effects from cortisol on nutritional status and cognition.

We conclude that cognitive disturbance is associated with low phosphate plasma due to protein malnutrition in patients at the time of diagnosis of PD (Fig. 1B). The results reveal the importance of including both phosphate and cortisol in future studies that examine nutritional status, cognition, and motor function in PD and for establishing new knowledge about mitochondrial energy production in the brain (Heinze et al., 2015). Low levels of plasma phosphate have never before been discussed as a metabolic marker and related to disease symptoms in the PD syndrome. A negative phosphate balance either due to low intake or losses due to stress and high cortisol may have multiple consequences for the disease pathogenesis of early PD.

## Sources of Funding

This work was supported by grants from The Swedish Parkinson Foundation (Svenska Parkinsonstiftelsen), Swedish Parkinson Foundation, Neuro Sweden, and Västerbotten County Council (ALF).

## Conflict of Interest

The authors report no disclosures relevant to the manuscript.
